# Supplementary material for: Outcomes of a QST Protocol in Healthy Subjects and Chronic Pain Patients: A Controlled Clinical Trial
Source: Biomedicines. 2023 Mar 27;11(4):1023. doi: 10.3390/biomedicines11041023 (PMC10136083; doi:10.3390/biomedicines11041023)
Supplement: Supplementary file 1 [file biomedicines-11-01023-s001.zip › biomedicines-2272084-supplementary.pdf]

Pain history questionnaire – English version

Date: __/__/__   Anonymization nº: ____   Sex __________   Birth date: __/__/__

A Brief pain inventory-based questions

1. Throughout our lives, most of us have had pain from time to time (such as minor headaches, sprains, and toothaches). Have you had pain other than these everyday kinds of pain in the last week?
2. If yes, for how long (months)? ________
   If your answer to question 1 was "Yes", please continue to answer the questionnaire.
3. On the diagram, shade in the areas where you feel pain. Put an X on the area that hurts the most.

"Human image: front and back"

4. Are you feeling pain right now?

4.1. If yes, for healthy subjects, we will perform the tests on another time.

4.2. If yes, for chronic pain patients, please rate your pain in a 0-10 scale (0- No pain and 10- Pain as bad as you can imagine) that best describes your pain at this moment.

5. For chronic pain patients, please rate your pain in a 0-10 scale (0- No pain and 10- Pain as bad as you can imagine) that best describes your pain at its minimum.
6. For chronic pain patients, please rate your pain in a 0-10 scale (0- No pain and 10- Pain as bad as you can imagine) that best describes your pain on the average.
7. For chronic pain patients, please rate your pain in a 0-10 scale (0- No pain and 10- Pain as bad as you can imagine) that best describes your pain at its maximum.
8. What treatments or medication do you do to manage pain?
9. During the past week, pain has interfered with your (rate your pain interference in a 0-10 scale: 0-Did not interfere and 10-Completely interfered):

9.1. General Activity

9.2. Humour

9.3. Sleep

B. Brief psychological status evaluation

1. Do you consider yourself a depressed person?
2. If you consider yourself to be depressed, are you taking any medication for depression?
